# The role of gender in patient preference for breast surgical care – a comment on equality

**DOI:** 10.1186/s13584-018-0231-2

**Published:** 2018-07-09

**Authors:** Tulin D. Cil, Alexandra M. Easson

**Affiliations:** 1 Department of Surgery, University of Toronto, Toronto, ON Canada; 2 Division of General Surgery, University Health Network, Toronto, ON Canada; 3 Department of Surgery, Women’s College Hospital, Toronto, ON Canada; 4 Division of General Surgery, Mount Sinai Hospital, Toronto, ON Canada

**Keywords:** Gender bias, Women in surgery, Breast surgery, Patient centered care, Patient preference

## Abstract

Gender preference among patients seeking medical care is an issue that is not well understood. It warrants exploration, particularly for patients undergoing sensitive physical exams. In a recent IJHPR article, Groutz et al. reported a survey study that explored patient preferences in selecting a breast surgeon. They found that a third of patients preferred a female surgeon for their breast examination. However, surgical ability was the primary factor in selecting a surgeon for their breast surgery. This commentary discusses these findings in the context of patient-centered care and issues of gender equality in medical education.

Gender equality is considered an important societal movement in achieving human rights for everyone based on their ability, rather than their gender and opportunity. This commentary argues that the goal of gender equality is why women should be encouraged to enter surgical professions, recognizing that patient preferences will be shaped by societal norms. Gender preferences for the performance of sensitive physical examinations by some patients are likely multifactorial and they warrant more exploration to deliver ideal patient centered care.

*“Gender parity at the United Nations is an urgent need and a personal priority. It's a moral duty and an operational necessity. The meaningful inclusion of women in decision-making increases effectiveness and productivity, brings new perspectives and solutions to the table, unlocks greater resources, and strengthens efforts across all the three pillars of our work.”*

- United Nations Secretary-General António Guterres

The United Nations has identified gender parity as an important mandate for the organization [1] . This goal is not only about increasing the quantity of women in its employ. It is the hope of shifting culture to create a society in which all women and men enjoy the same opportunities, rights and obligations in all spheres of life. Gender equality is intrinsically linked to sustainable development and is vital to the realization of human rights for all. In such a society, all people will have the same opportunities to find the roles and professions that best fit their unique talents, allowing them to excel.

Medicine is a profession where its members are expected to be among the most highly skilled in society. It is perhaps not surprising then that gender parity in medicine has been an evolving issue. We have seen increasing numbers of women entering medical school – in some jurisdictions this proportion is higher than their male counterparts. In many specialties, however, women are still the minority. Furthermore, equity in numbers has not translated to equality in roles, nor in the achievement of leadership positions [2]. This is particularly true for surgery and its subspecialties.

This commentary is based on the recent paper by Groutz et al. [3] on gender preferences for patients undergoing breast surgery consultation. The study topic encompasses the domains of patient centered care and patient-preference, gendered roles and bias surrounding these roles, and the issues of women in medicine, particularly surgery.

The authors performed a survey to assess gender preferences of women regarding their choice of a breast surgeon. Five hundred women at 2 tertiary care hospitals responded to the 25-item questionnaire, which focused on the role of physician gender in the provision of breast exams and breast surgery. The authors found that one-third of women preferred a female breast surgeon for the clinical breast exam (CBE) due to embarrassment in a sensitive exam setting. Most significantly, however, 73% of respondents had no gender preference regarding performance of breast surgery. The most important factors for patients when choosing a surgeon were ability, experience and knowledge.

This phenomenon is not unique to breast surgery. Several studies in other medical disciplines have highlighted the role of gender in patient preferences. Similar research from Groutz et al. [4] assessed the issue of gender for patients when seeing a urologist. Many (43%) of the male patients expressed a preference for a male urologist for both the physical exam and surgery. Plunkett et al. [5] examined patient preference for gender concordance in obstetrics and gynecology. Over half of the patients in that survey study preferred a woman OBGYN; at the same time, factors such as bedside manner, hospital affiliation and recommendations from other physicians and friends/family were more important than gender. A recent plastic surgery study found most patients (46% of 200 women surveyed) did not request a woman surgeon if given the choice; however, of those having breast and face surgery 25% were interested in having a woman surgeon [6]. This suggests that for some patients who need to have sensitive examinations, such as breast, urology and gynecology, gender concordance with the surgeon may be a relevant factor.

Certainly the ability to have patient preference-based care, as exemplified in shared decision-making, has been shown to be valuable, with patients expressing high satisfaction with such approaches [7]. Some preferences however may also have an impact on the use of health services and further exacerbate pre-existing health care disparities. Could the issue of surgeon gender result in a patient not seeking care from the onset? There is some data to suggest that patients may forgo necessary procedures, such as colonoscopy, based on the gender of the health care providers. If this were to be the case with breast exams, perhaps some patients might avoid a consultation for a necessary clinical concern.

The question remains as to why patients may declare such preferences. The current study suggests that the main reason is a patient feeling of increased embarrassment when undergoing a sensitive physical exam. Patients who were younger and those that were married expressed the most preference for gender concordance. Several studies have examined the practice patterns of male and female physicians. A 1997 Dutch Study [8] evaluated patient preference for genders of multiple different health professionals. The authors found that if patients preferred a female health professional, it was not only that they felt more at ease with sensitive exams by females but they also indicated women physicians were easier talk to. More than twenty years later, these findings persist in more recent data regarding communication styles of physicians [9].

Societal norms can shape how patients may view physicians in various specialties. A study by Dusch et al. [10] found that while patients did not indicate a preference based on gender per se, they indicated they would select a thoracic surgeon that displayed a more masculine presence and was firm whereas a breast surgeon that was more communal and nurturing would be ideal. The authors hypothesized that although their patient population evaluated surgeons based on competence rather than gender, women were still thought to possess a more communal, or more feminine, attitude (given the psychology of a breast cancer diagnosis). Furthermore, they speculated that perhaps breast surgery was perceived as less technically complex than thoracic surgery, requiring a less aggressive surgical approach.

## Conclusion

Groutz et al. [3] argue that the response to some patient’s preference for a female breast surgeon is to increase the number of women in surgery. They highlight a number of methods whereby this can be achieved, such as increasing exposure of medical students to surgery as a career, and promoting female-friendly working conditions. While we agree with these suggestions, we caution that this must be done without further reinforcing societal stereotypes of men and women. Increasing the number of women in surgery is part of an important movement to achieve equal human rights for all, based primarily on one’s abilities. Addressing the gender gap in surgery is an active process that is multifaceted and must take into account barriers and enablers that have served to keep women as the minority in surgical training.

The understanding that some patients will prefer a gender concordant physician for certain aspects of their medical care is important when delivering patient centered care and should be respected if possible, for men and women alike. Gender preferences for the performance of sensitive physical examinations by some patients are likely multifactorial and warrants more exploration to deliver ideal patient centered care. Asking patients about their feelings, asking permission to perform an examination or offering an explanation of why the examination is important, and if possible, providing a gender concordant assistant for that portion of the examination, are all potential strategies that can be explored. When these factors are better understood, it may be possible to have caring interactions with patients about sensitive physical exams, and then the gender of the surgeon will hopefully play a small or at least smaller role in patient-physician relationship.
